# A Theoretical Framework to Assess the Impact of Flooding on Dairy Cattle Farms: Identification of Direct Damage from an Animal Welfare Perspective

**DOI:** 10.3390/ani11061586

**Published:** 2021-05-28

**Authors:** Anna Gaviglio, Annafrancesca Corradini, Maria Elena Marescotti, Eugenio Demartini, Rosalia Filippini

**Affiliations:** Animal Science and Food Safety (VESPA), Department of Health, University of Milan, 26900 Lodi, Italy; anna.gaviglio@unimi.it (A.G.); maria.marescotti@unimi.it (M.E.M.); eugenio.demartini@unimi.it (E.D.); rosalia.filippini@unimi.it (R.F.)

**Keywords:** flood impact, rural appraisal, economic damage, livestock, climate change

## Abstract

**Simple Summary:**

Identifying the damage caused by extreme weather events due to the climate change phenomena to formulate targeted coping strategies and policies is an issue that concerns all economics sectors. To date, the impact of flooding events on agriculture, particularly livestock, has been minimally investigated. For this reason, this study seeks to identify flood damage to livestock production, focusing in particular on dairy cattle farms. In fact, the herd is the main source of income for dairy farmers and the impact of flood on dairy farm can lead to several problems that can seriously undermine dairy cattle welfare and consequently the production outcome after a flood event. Therefore, this study identified and quantified the flood damage that may affect dairy herds, as reported in the literature. This study might help the development of a strategy able to assess direct damages to livestock welfare caused by flood events, provide advantages for farm management and contribute to farm resilience after a natural disaster.

**Abstract:**

For the economic sectors, the need to address the challenges posed by natural disasters due to climate change is an outstanding issue. To date, according to the European Commission (2019), there is still a gap in the estimation of the costs of flood in all European countries and the direct impact that these floods have on agricultural activities. More specifically, the damage to livestock has been minimally studied. The aim of this study is is therefore to identify the flood damage that affects dairy cattle farms, focusing on the damage to herds caused by a flood event; in fact, poor welfare conditions of dairy cattle directly affect production and thus farm revenue. To accomplish the aim of this study, a framework was first developed to identify possible damage types. Then, scientific literature focusing on the identification of flood damage to dairy herds was reviewed, and to quantify this damage to herds, literature sources providing information on the magnitude of variation in the identified damage types were used. Thus, our results provide relevant information on the variables that should be taken into account when assessing of the direct damage affecting the overall welfare of a dairy herd after a flood event. This evidence could then contribute to the development of tools aimed at assessing damage to dairy cattle on flood-affected farms.

## 1. Introduction

The debate on climate change has raised several concerns related to the damage caused by natural disasters, which influence the development of human society [1]. In particular, the effect of flooding on agriculture is relevant since it directly affects the food security and food safety of people around the world [2].

Worldwide, between 1995 and 2015, flood events accounted for 47% of all weather-related disasters and caused approximately 25% of the total economic damage due to natural disasters [3]. Nevertheless, according to international reports, the true economic cost of weather-related disasters could be worse than the official figures, since only 35% of records include information about economic losses [3]. In European countries, between 2010 and 2016, at least 128 damaging flood events occurred, and almost half were registered in only four countries, namely Italy (22%), France (12%), Spain (1%) and Germany (9%) [4]. Paprotny et al. (2018) [4] also show that although flood events are increasingly small in terms of severity, their increasing occurrence causes more damage over longer periods.

For this reason, the European Union developed European Directive 2007/60/EC, also known as the Floods Directive (FD). The directive aims to establish a reference framework for the assessment and management of flood risk in the European Union [5]. The main purpose is to reduce the negative consequences of floods on human health, economic activities, the environment and cultural heritage. To address these goals, the FD outlines an implementation path through which each member state shall draft appropriate flood risk management plans (FRMPs). Such plans have the objective of defining the measures that need to be applied to mitigate the potential damage of the floods to the areas at risk. Estimating the economic damage caused by floods is essential to designing appropriate restoration measures for these plans.

Nevertheless, based on the evaluation of the first FD programming cycle (2010–2015), in European countries, only 10% of the measures were focused on recovery actions [6]. According to the European Commission (2019) [6], there is still a gap in the precise estimate of the costs of floods in all the European countries. Thus, more research should be conducted to estimate recovery actions and the related costs resulting from a flood event.

Among the economic sectors, agriculture has received less attention than other sectors [7,8] and currently, most of the literature focuses mainly on cropping systems rather than on livestock production [9].

Given this context, the present study proposes a comprehensive methodological framework of damage categories that should be considered when evaluating flooding impacts.

The hypothesis of this study is that: (1) when flooding occurs, a farmer is more interested in recovering economic activity than closing the farm; (2) when a flood event occurs, there is a decrease in dairy welfare, which may lead to production losses. Thus, the research aims to identify the variables that can be used to estimate the economic damage that livestock farms may experience after flooding (the analysis proposed here is part of a broader research project that aims to estimate the economic damage of floods experienced by livestock farms. This study follows the first methodological study developed with the purpose of featuring all the damage that a dairy farm may face after a flood event. This first study identified all the connections between the damage to the different elements of a livestock farm (i.e., buildings, herd, machinery, feed and roads) to provide a systemic framework of the overall dynamics). To do accomplish this goal, we first identify the damage to the herd caused by flood events through an analysis of the available literature on the topic. By achieving its objective, this paper will be instrumental in future estimations of the increased costs and decreased revenues resulting from flood events. The remainder of the article is organized as follows. The next section, Theoretical Background, provides a deeper understanding of flooding damage, especially dairy farming. In the subsequent sections, the methodology and results are described and discussed.

## 2. Theoretical Background

### Flooding Damage to Livestock Production: A Case Study of Dairy Cattle Herds

According to Merz et al. (2010) [10], there are two kinds of flood damage: direct and indirect. Direct damage is the damage caused directly from the physical contact of floodwater with humans or other objects. Indirect damage occurs to elements not directly exposed to the flood water but connected to the other elements that experienced direct damage, and for this reason, this damage may occur outside of the flood event [10]. In the literature that explores flooding impacts on agriculture, direct damage can include the destruction of crops, decreased quality of the product and damage to soil due to pollutants or soil erosion [8,11,12]; other studies expand the scope of direct flooding damage to buildings and infrastructure, considering the cost of cleaning and evacuating [10,13,14]. Indirect damage can be the disruption of economic activity due to the loss of production. Nevertheless, the interpretation of indirect damage may vary depending on the purpose of the study. For some scholars, direct damage is the economic loss (i.e., Forster et al., 2008 [11]), while for others, direct damages are just the loss of the yield with which the economic consequences are associated (i.e., Hussain, 1995 [15]).

The literature recognizes the presence of macroeconomic damages as short- or long-term impacts on the economy (i.e., Parker, 2004 [16]). Merz et al. (2010) [10] also distinguish also between micro-, meso- and macro- spatial scales in the analysis of flood-related damage. In micro-scale assessments, the assessment is conducted on every single element of the economic activity, i.e., buildings or infrastructure objects. At the meso-scale, the assessment is based on the spatial aggregations of the elements; thus, they are conducted, for example, at the scale of residential areas. Finally, in the macro-scale damage assessments, the analysis is based on large-scale spatial units, such as municipalities, regions and countries. The temporal dimensions of these assessment are another crucial element since the temporal scale defines how many effects should be taken into account in a model and thus the magnitude of the flood damage [10].

In broad terms, livestock farms are complex interdependent systems, especially dairy farms [9]. A dairy cattle farm is a system based on the interaction of different integrated elements: the farm’s crops and supplementary feed, the capital assets in terms of buildings and structures, and off-farm support from infrastructure and rural service providers [17]. These factors should be harmonized with the dynamics of the local and global markets, both in the sale of outputs and in the acquisition of inputs.

Few studies to date have analyzed the economic damage of flooding on livestock farms; those that have been carried out, especially in developing countries, consider the damage to livestock a consequence of flooding, but the studies do not give the damage a dimension (e.g., the increased incidence of a certain disease as a consequence of the poor hygiene conditions of a farm after flooding is reported but without any value associated with its variation) [18,19]. Other studies in developed countries quantify the economic impact of flooding on livestock, but they do so from a territorial scale, thus comparing different areas in the same region [20,21]. Additionally, these studies do not provide details on the direct damage to the welfare of the livestock affected by floods. From a pragmatic perspective, detecting such damage should be the starting point for describing the flooding impact at the farm level. In fact, while the economic impacts in terms of increased costs and reduced revenues are linked to the specific place, region, and nation where the flood occurs, the damage to animals can be more similar looking at different regions and even to different production systems and breeds. In particular, focusing on dairy cattle farms, not only lactating cows but also the entire herd of dairy cattle represent the main source of revenue for the farm. Hence, in this case, understanding exactly what affects the animals’ experience is the first step in determining which recovery actions should be undertaken and thus estimating the economic impact of such recovery. Another element exacerbating the economic damage to livestock farms—and which make its estimation more complex—is the time of recovery after a flood event. In cropping system damage models, the recovery time is usually one year, corresponding to the recovery time of the field [11]. In the case of livestock, the recovery time determination should consider that one animal could be a source of revenue for a farmer for more than one year—in case of dairy farms, even five years. Thus, for dairy farms, the estimation of the damage to herds should follow the growth of the animals from birth to productive period, usually between two and three years. Gaviglio et al. (2019) [9], for example, reported that farmers envision that it will take at least three years before the preflood production level is reached; in fact, in three years, the calves born after the flood became productive. In particular, the authors reported several steps that a farmer needed to follow over time to restore farm activity. Farmers had to relocate livestock since the stables were unusable, and after one month, they could clean the farm. After approximately two months, the farmers were able to move the animals back. After that time, for approximately one year, farmers had to be very attentive to the problems that may occur in the herd, as they may be related to the flood and may therefore need specific investigations and treatments. After one year, the farmers could restore the animal diets with the new harvest. Thus, the farmers envisioned that at least three years were needed to reach the preflood production level (actually, the complete restoration of the herd may take longer, also considering genetics). Given that flood events may result in direct damage to an entire herd, recent literature has suggested a link between flood events and decreased levels of animal welfare [22,23,24]. In fact, natural disasters can be detrimental to animals both physically and emotionally. Consistent with this view, the guidelines for veterinary services proposed in 2016 by the World Organisation for Animal Health (OIE) (2016) state the need to improve disaster management and risk reduction in relation to animal health, animal welfare and veterinary public health [25]. Protecting animals during disasters and ensuring their basic welfare level during emergencies could be a challenging task to the extent that the impact may cause massive livestock losses. Moreover, according to scholars, to efficiently cope with flood damage, the priority is to provide livestock with adequate care after the emergency to limit future production losses and stem economic flood damage to the farm.

Animal welfare, however, is a complex concept, especially considering that despite the improvements made in farming in recent decades, animal welfare assessments on farms are still a debated issue [26,27]. Animal welfare involves the coexistence of several closely interconnected and mutually overlapping dimensions: biological functions, natural life and affective state [28]. All these dimensions of a dairy herd affected by flooding are presumed to be compromised to an extent, dependent on parameters of vulnerability (depending on farm characteristics, e.g., herd health status preflood) and hazard parameters (depending on flood characteristics, e.g., season in which the flood event occurs, speed of water and sediment), as proposed by Gaviglio et al. (2019) [9].

**Figure 1:**
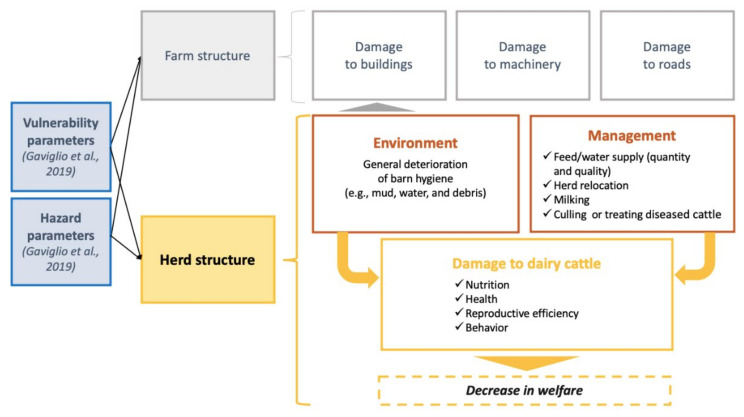
Framework for flooding damage to dairy cattle farms (expanded upon that in Gaviglio et al., 2019).

According to this assumption, we expanded upon the framework proposed by Gaviglio et al. (2019), focusing on direct damage to herds (Figure 1). Involving experts in the field (i.e., farmers and animal welfare technicians), different categories of damage to dairy herds affected by flooding were identified. As reported in Figure 1, “damage to dairy herds” includes four subcategories of damage that cause a decrease in animal welfare: nutrition, health, reproductive efficiency and behavior. Then, with the intention of taking a holistic approach to the issue, two categories referred to as “management” and “environment” were considered. In fact, these factors greatly influence the welfare status of livestock. This influence seems particularly true considering the “flooded farm” scenario as an extreme condition where the environment could be seriously compromised, and farmers’ management decisions play a vital role in the economic sustainability and resilience of the farm.

## 3. Materials and Methods

**Figure 2:**
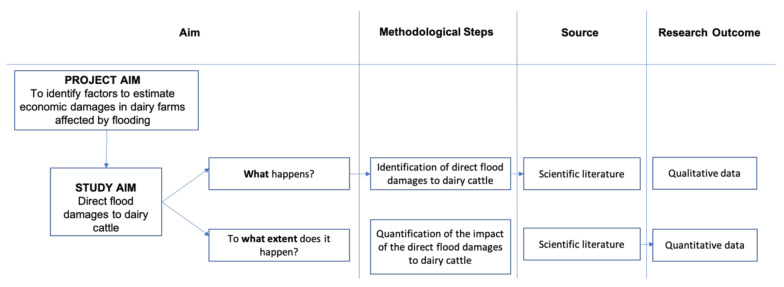
Purpose of the study and methodology.

To achieve the research objective, i.e., to identify and provide dimensions for the direct damage from flooding to dairy cattle herds as they relate to dairy cattle welfare, the analysis of the literature was divided into two steps (Figure 2). The first step focused on the identification of flooding damage to dairy herds already observed in the literature, either based on case study analysis or theoretical hypotheses, to qualitatively determine what the impacts are on dairy herds when floods occur. The second step focused on the quantification of the impact of any identified flood damage. The search was performed using two of the main scientific databases, namely Scopus^®^ and Web of Science^®^.

### 3.1. Identification of Direct Flood Damage to Dairy Cattle Herds

**Figure 3:**
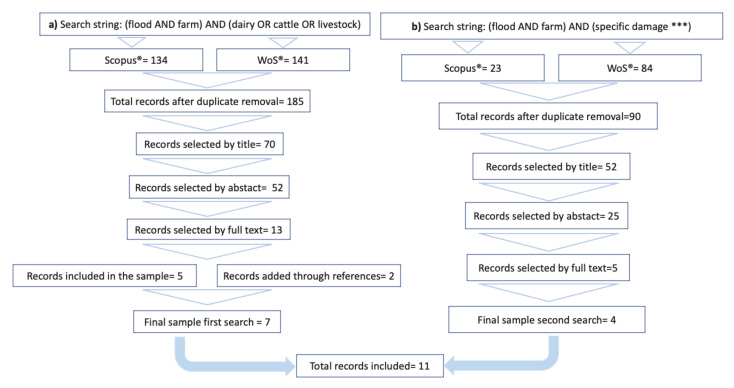
(**a**,**b**) Selection of literature from Scopus and Web of Science: first search identified the type of damage (**a**) on the left, and (**b**) the second search quantified the specific damage types on the right. *** specific damage types: “flood” and “cattle” or “dairy” and “lameness”; “Clostridium” or “*Fasciola hepatica*” or “anthrax” or “leptospirosis”; “reproduction” or “abortion” or “conception rate”; “feed supply” or “water supply”.

To identify the literature that evaluates flood damage to herds, we applied the following approach. The first literature search was performed in databases Scopus^®^ and Web of Science^®^, which contain peer-reviewed material. For each database, we used the same search strings based on the keywords “livestock”, “cattle” and “farm” combined with the words “flood” and “farm” (Figure 3). No timespan was set on the search, but only papers written in English were selected. The inclusion criteria for article selection referred to the identification or description of direct impacts on cattle management, cattle production and dairy cattle welfare from floods, including secondary studies due to the limited resources available on the topic.

Finally, selected papers identified and/or described direct damage to dairy herds caused by floods in inland areas.

### 3.2. Dimensions of the Impacts of Direct Flood Damage to Herds

The analysis was expanded, with the intent of linking a damage dimension of the impact to the damage identified through the literature reviewed. Thus, (1) damage types were identified in the seven selected papers (3.1); (2) for each type of damage detected, a second literature search was conducted using Scopus^®^ and Web of Science^®^.

The specific literature was therefore used as an instrument to identify the magnitude of the change in animal conditions that occurs when a flood dramatically alters the environmental conditions of a farm. The search strings used to identify the impact dimensions of each issue were based on the keywords “flood” and “cattle” or “dairy” combined with the term denoting the specific type of damage (e.g., “flood” and “cattle” or “dairy” and “mastitis OR somatic cell count”). No timespan was set, and only peer reviewed articles written in English were selected. The number of records that were found in the databases, excluding duplicates, was 90. The papers were then reviewed; the inclusion condition was that data were provided about the impact of the flood on cattle and referred to the specific damage.

The final sample of literature contained seven papers retrieved from the first search and four retrieved from the second search.

## 4. Results

Overall, most of the papers that analyze direct damage to a herd from a flood were published after 2010 (Table 1). In particular, seven of the eleven were published from 2018 to 2020. This finding is consistent with the fact that flood occurrence has become more frequent in the past decade, and its detection is becoming a relevant issue in the scientific community. Moreover, the systematic inclusion and evaluation of direct damage to herds occurred especially in the United States and Europe, while three papers were focused on Asia. Finally, four papers out of the eleven included surveys and stakeholder focus groups as the basis of the methodology, again demonstrating the lack of case study analysis on direct damage to livestock.

### 4.1. Identification of the Direct Flood Damages to Herd

Table 2 shows the main variables identified by the selected literature. For these variables, categorization was performed to properly understand the recovery time of the herd.

**Figure 4:**
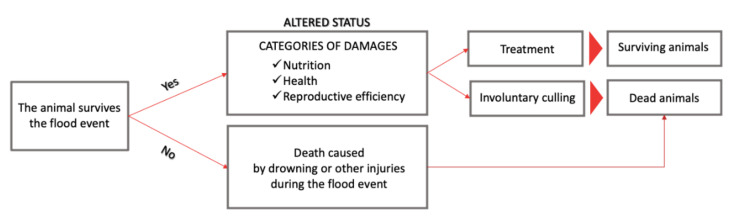
Categorization of variables.

As reported in Figure 4, a first level of categorization divides the damage to the herd considering that on the one hand, in the worst case scenario (depending on the vulnerability and hazard parameters as defined by Gaviglio et al. (2019)) [9], the animal may not survive the flood event or it must be culled by the farmer, and the on the other hand, in most cases, the animal that survives the flood, but may need to be treated by the farmer (Figure 3). A second level of categorization describes in more detail what happens to the herd (Figure 2). Health issues and problems related to reproductive efficiency may be factors that lead a farmer to the decision to cull an animal. Given the extraordinary nature of the event, this management decision may therefore not follow principles primarily related to profit maximization. Thus, it can be assumed that a farmer’s decision may be to treat a larger number of animals than would be treated under normal conditions; thus, the farmer is making choices that take into account, in a narrower sense, the cost-benefit ratio relating to the recovery of a level of productivity sufficient to not go out of business. Livestock can also drown during a flood. Even if livestock do not drown, the herd can still have health and reproductive issues and problems related to feeding that can undermine revenue generation in terms of increasing costs and decreasing revenues.

In addition to the damage that impacts the herd in the time period that follows the flood, it is essential to consider the damages that impact the herd at later times. For this reason, three timespans were identified (Table 2). First, a short period of approximately two months is necessary for a farmer to restore buildings and machinery and to be able to move animals back into the barn. Even when livestock are not relocated, livestock may experience a compromised environment, and may need time to recover. Second, a mid-term period of approximately one year is necessary for a farmer to restore the livestock diet with his or her own crops. Third, there is a long-term period in which a farmer could restore his or her dairy farm to the pre-flood level of production.

The following subsections describe the consequences of flood events on animals. Sub-section *a* considers the consequences on the surviving animals in terms of health (a.1), stress (a.2), malnutrition (a.3) and reproductive efficiency (a.4). Sub-section *b* takes into consideration the dead animals in terms of involuntary culling causes and other causes of death due to the flood event, namely, health problems (b.1), drowning (b.2) and reproductive efficiency (b.3).

(a). Surviving animals

When the animals survive, they must cope with an altered environment, a possible malnutrition in terms of the quality and quantity of water and food, and reproductive problems. If the animals relocated to other places, then contamination with other herds may undermine the health of the cows. If the animals are not relocated, then, the flood alters the spaces where the livestock is placed, causing health problems. According to the literature, farmers have experienced the occurrence of the same typical diseases after a flood event but in greater amounts and for a longer period.

(a.1). Health

- *Mastitis*. In the after-flood farm scenario, environmental conditions are considered by the literature as triggers that increase mastitis in a herd. In the short term, muddy and the wet environmental conditions could facilitate the proliferation of environmental pathogens responsible for mastitis. According to the studies, farmers report that during the first year after a flood, the occurrence of mastitis continues to be at higher levels than normal. Additionally, the change in the diet that a farmer needs to adopt if the food stock was compromised could contribute to the weakening of the immune system, predisposing the animals to a greater risk of infections.
- *Lameness.* Lameness could arise after a flood event due to the muddy and wet conditions. In fact, interdigital skin and hooves could soften due to the adverse environment. Lameness is considered to be a problem linked to several risk factors for which hygiene management of the farm environment plays a crucial role. In the studies, as in other bacterial diseases, farmers detected a more frequent occurrence immediately after the flood. The predisposition to higher lameness occurred throughout the first year.
- *Bacterial and parasitic diseases*. The risk of infections causing bacterial diseases was reported by farms affected by floods. In particular, clostridial diseases (*Clostridium septicum*, *Clostridium haemolyticum, Clostridium chauvoei, Clostridium perfringens and Clostridium tetani*) and leptospirosis (*Leptospira* sp.) can become a serious issue threatening the health of a herd after a flood event; the risk of an anthrax infection was also reported. Moreover, the risk of parasitosis, caused by both external and internal parasites was documented; more specifically, the highly moist condition of the soil is considered as a predisposing factor for liver flukes (*Fasciola hepatica* in particular), which was reported to be mainly related to grazing herds.
- *Injuries.* The most common injuries identified by literature were fractures, open cuts, wounds and devitalized tissues. These injuries were reported as immediate damage to herd health status, thus happening especially in the short-term because of contact with water during floods. The causes of these injuries were especially related to the need to relocate the livestock but also to the permanent damages resulting in unhealthy and wet environments. According to scholars, it is important to consider these injuries in the evaluation of the damage to a farm since beyond the costs of recovery, they may also predispose the livestock to infection, which can result in a lethal outcome.

(a.2). Stress

Although the literature does not distinguish between acute and chronic flood-related stress, general considerations regarding the negative implications of stress on the immune systems of cattle have been reported. Although the literature does not provide a precise time span for the impacts, it can be presumed that flooding may generate a short-term and a long-term impact on cattle; in certain cases, the physiological balance may be fully recovered quickly, while in other cases (chronic stress), a maladaptive response may occur, significantly impacting the future welfare and productivity of cattle, even after emergency strategies to cope with the disaster impact have been adopted.

(a.3). Malnutrition

Feeding and watering dairy cattle after a flood event is a serious issue related to animal welfare status. In fact, studies that aim to identify damage due to flooding report that problems may arise in satisfying all animals’ needs, considering the high contamination risk for feed and water supplies.

- *Feeding.* Floodwater may heavily affect the quality of feed supply stocked on farms; contamination caused by toxic mold growth and the presence of foreign material and debris may severely endanger dairy cattle health. According to studies based on farmers’ surveys, the feeding damage impacts a herd for the first year. The flood may impact the silos where the feed is stocked. In the first year, farmers must change the diet of the animal, trying to replace the feed ration with what is possible to find at the market. It takes one year to harvest the new field crops and thus recover the original diet. It is important to consider the diet change in estimating the economic damage since beyond the costs of replacing feed, challenges to feed access, poor feed quality and prolonged stress could act as predisposing factors to metabolic disorders, which can impact livestock production and for which prevention is needed. Furthermore, the change in diet could contribute in the short-term to a decrease in milk yield, resulting in loss of revenue.
- *Watering*. Water supply contamination can occur as a consequence of floods; floodwater could carry debris or chemical or physical hazards and may also be contaminated by harmful bacteria and viruses. Studies have also identified that farmers criticized temporary disruptions to potable water supplies, incurring additional costs to secure water for their livestock. To prevent animals from drinking contaminated water, providing them clean water in an adequate quantity according to their vital and productive stages must be a priority immediately after a flood event. At the same time, even in the case that the water provided in the stable is not contaminated, when animals are relocated to other structures in the short-term, we can assume an increasing cost of water provision.

(a.4). Reproductive efficiency

Reproductive problems can occur as an effect of flood events; stress, malnutrition, diseases and poor general health conditions may lead to early miscarriages for pregnant cattle and to conception difficulties for non-pregnant cattle and heifers. This condition may disrupt the reproductive plan (e.g., increase in the number of days open), provoking relevant production issues.

(b). Dead animals: causes of involuntary culling and other causes of death due to a flood event

The death of dairy cows was noted by several authors. Several causes that were identified included drowning during floods and issues related to health and reproduction, which may have led farmers to decide to cull the cattle. Table 2 shows that this damage can occur in over both short and medium periods. The impact over the long-term may depend on the decision of a farmer to replace the culled cattle. If the farmer does not decide to replace livestock, then the scientific literature has estimated an impact on the production of approximately three years (Table 2). In three years, the calves born after the flood become productive, and the farm could reach its pre-flood production levels.

(b.1). Health

The decision to cull or treat animals that are injured or sick mainly depends on the severity of the injuries and diseases. Among the injuries, foot and leg fractures are the most crucial in determining if the injury is worth treating or culling livestock, and this decision must be made in the immediate post-flood period, when most of the injuries connected to a flood occur. Moreover, open wounds resulting from floods may facilitate the spread of bacterial diseases and thus cause death. Floods compromise the health of livestock for at least the first year, causing inexplicable deaths. According to farmers’ surveys during the first year after a flood, the death of animals is more frequent and sudden than usual due to health issues. Scholars have hypothesized that for all the inexplicable deaths, it could be interesting to perform necropsy to determine the causes of death.

(b.2). Drowning

According to the literature, few farmers have experienced animals drowning or contracting aspiration pneumonia during flooding. In fact, farmers immediately take action to save the animals, especially on dairy farms, where lactating cows are the main source of revenue for the farms. Drowning can be considered a short-term type of damage, even though the farmer can decide, for example, to replace the animal that dies by buying another animal at the same productive stage or, if the animal that dies is not a calf but a heifer or a cow, to wait for the female calves already present in the farm to become productive. This situation may have long-term effects on the herd, considering its complete restoration.

(b.3). Reproductive efficiency

Miscarriages can happen as direct consequences of floods, but also as indirect consequences such as from damage to buildings and other structures. Typically, depending on the stage of pregnancy, a farmer can decide to cull or to maintain a cow for a future insemination. Usually, when miscarriages occur late in gestation—at least 7–8 months into a cow pregnancy—the solution is to cull the animal. The reason for this is the potential lack of production, which depends on cow maternity. According to farmers’ surveys during the first year, reproductive problems occurred for at least the first year after a flood; thus, flood events also affect the loss of animals due to reproduction-related causes.

### 4.2. Quantification of the Variation in The Direct Flood Damage Types Affecting Herds

Table 3 includes the quantification of the identified types of direct damage to herds after a flood event. The table is based on the assumption that when a flood occurs, there is a decrease in the general animal welfare of a herd. The first concern of a farmer is to treat the animals and restore animal welfare to recover the level of production. Thus, the literature regarding the specific identified direct damage types was studied and where it was not possible to find information, an approximation related to poor barn hygiene conditions was applied. Table 3 also includes whether the data provided in the literature referred to the short-term (2 months) or medium-term (1 year).

The deteriorated hygiene status of a farm after a flood and, in general, the impact of flooding on the environment, could be responsible for the onset of new diseases or the exacerbation of some health issues already present within a herd. In particular, as reported in the literature, an anthrax outbreak apparently related to flood events that occurred in grazing cattle was reported in southwestern Bosnia and Herzegovina in 2010, highlighting the potential for increasing outbreaks in areas where this disease occurs sporadically [34]. Regarding clostridial diseases, a study conducted in Taiwan in 2013 found significant changes in the environmental distribution of *Clostridium* spp. after flooding on farms [36]. Furthermore, two recent studies have reported the seroprevalence of leptospirosis in cattle after flooding; in particular, a study conducted in Malaysia published in 2020 found a significant association between livestock (sample of 1024 cattle, 366 goats and 338 sheep) that were exposed to floods and leptospirosis seropositivity, highlighting that livestock exposed to floods were 2.7 times more likely to acquire leptospiral infections than livestock that were kept under safe conditions (Table 3).

Furthermore, several studies have investigated the relationship between dairy hygiene and somatic cell count; in fact, present evidence suggests a strong correlation between udder cleanliness and chances of developing environmental mastitis. More specifically, a study conducted on 1250 lactating cows during a period equal to one lactation (approximately 1 year) reported that cows with dirtier udders are 1.5 times more likely to have major pathogens isolated in milk samples (Table 3).

The prevalence of lameness seems linked with a poor level of animal care and poor hygiene; these are in fact predisposing factors that can increase the risk of lameness. One of the latest studies conducted on 18 Canadian dairy herds reported that cows housed on farms with dirtier stalls were 1.3 times more likely to be lame, over the time of the observation.

The mastitis and lameness values were reported for one-year observations (Table 3). Thus, it is not possible to have an estimate of the short-term period for the flood model. It is also true that in the case of floods, the emergency period can lead farmers to make different management decisions. Animals can be moved to a different area of the farm or relocated to another farm. In both cases, we can assume that the occurrence of health issues (in terms of reproductive efficiency, lameness and mastitis-related damage) can be greater in those locations than the original location. In the first case, spaces could be inadequate and environmental conditions could be adverse. In the second case, it can be argued that under flooded farm conditions, the classic rules of biosecurity may not be respected due to the nature of the emergency. As a general rule, livestock that are housed in a stable other than that of origin should be subjected to a period of quarantine to avoid precarious situations that may predispose herds to infectious agents.

In terms of reproductive efficiency-related damage, no quantitative data were found in the literature about the variation in reproductive variables when hygiene or the farm environment and management changed, considering specific literature on dimension quantification and literature on floods.

Finally, considering nutrition, the measures were obtained from scientific literature on the flood. The literature on floods notes that in the worst-case scenario, in the first year, all the feed could be eliminated since flood can spoil trenches and silos. Preventing this scenario seems to be essential for avoiding animal welfare issues that could also affect future production caused by compromised or contaminated feed. Thus, a complete change in livestock diet seems to be a plausible option. This option seems also relevant to water supply. In fact, several studies have observed that sources of water supply or on-farm water reserves may need to be completely replaced in the short-term. This happens when the water is contaminated and/or the cows need to be relocated.

## 5. Discussion and Conclusions

To date, in comparison to other sectors, the agricultural sector has been less covered by studies about the economic damage of floods. According to Merz et al. (2010) [10], this finding can probably be related to the fact that the economic damage from floods to the agricultural sector is considered lower than that to other economic sectors in urban areas. Farmers consider the adoption of insurance policies too expensive to protect against losses of capital, production, machinery and plants in the case of natural calamities, such as flood events [40].

This study focused on the micro-scale [10] to detect the direct damage to the herds. The literature review on flood impacts showed that while some types of flood damage is well documented, such as mastitis and malnutrition, other types of damage are less documented. This scenario can depend on the greater occurrence and the certainty of the economic impact of specific diseases. For example, mastitis is one of the most common and expensive diseases in the dairy sector, [41] and it can be a relevant issue to manage on a dairy farm after a flood event. In contrast, according to the literature, lameness is usually underestimated under normal conditions [42] and thus is even more difficult to detect under extraordinary conditions such as those associated with natural disasters. Furthermore, with regard to diseases that appear to be closely linked to flooding, such as detected cases of leptospirosis [30,33,35], even though its prevalence is mostly reported in countries where this disease represents a more relevant problem than in Europe in terms of cases, an increase in cost linked to its management on farms after flooding is expected. Considering this particular case of leptospirosis, both the possible increase in the use of post-exposure medication (if animals are not vaccinated) and the risk that leptospirosis infection may lead to abortion in pregnant cows and heifers may negatively affect farm finances.

The relative importance of certain types of damage over others in the literature can also depend on the fact that few case study analyses have been conducted on the topic; thus, an increasing number of studies about what happens on a farm after a flood should be carried out. In fact, as far as we know, there are currently no comprehensive studies that aim to quantify the extent of flooding damages to dairy farms in economic terms. As Paulik et al. (2021) [17] note, the difficulties in estimating flood damage to livestock farms are connected to the challenges of accessing farms, the long-term emergence and accumulation of losses and the ethical concerns relating to carrying out research during disaster recovery.

Considering the lack of information, this study included papers addressing not only dairy farms but also beef cattle farms to obtain a wider perspective on the damage already investigated in the literature. Then, the studies focusing on damage more connected to dairy farms were selected.

The lack of data on damage leads to a lack of understanding of how great the impact is. The quantification of the damage is essential to estimating the economic damages of a flood on a herd; thus, in this study, specific literature on animal health, such as Schreiner and Ruegg (2003) [38] and Robles et al. (2021) [39], were used as instruments to understand to what extent damage occurs when the environmental conditions of a farm change. Nevertheless, it is difficult from such literature to determine an economic impact since the studies refer to different temporal spans, use different units of measure and refer to specific case studies. Given this evidence, limitations of the study relate first to the few results that could be provided regarding the quantitative impact of flooding on dairy cattle and second to the difficulty of retrieving studies related to similar conditions (i.e., poor barn hygiene status). Furthermore, future studies should consider more in depth that animal categories present on a farm (calves, heifers, non-lactating, early lactation, late lactation cows) may be affected differently from a flood event and that this may also lead to different impact on their production outcome.

Estimates of the economic impact of diseases have been determined [43,44], but these data refer to estimates under normal conditions, while in the case of flood damage, it is relevant to understand what changes and to what extent the ordinary situation changes. Moreover, studies do not properly distinguish between implicit and explicit costs. The economic impact of the diseases should consider the costs of treatment, as well as the loss of revenue from the treatment days (approximately ten days) and the waiting days (from three to six days). The economic effect impact also depends on the contingent situation of the farm: how many cattle are diseased, for how many days is the milk is not marketable, what is the quality of milk and what is the price of milk.

All studies on floods have highlighted the need for a change in diet in the first year, especially if floods occur after the harvesting season. The economic impact is connected to the need to change the livestock diet to ensure the same production level. The provision of the appropriate feed could be affected by the availability and profitability of crops at the markets. If the appropriate crops are not available, then farmers may be required to provide a feed that does not ensure the same levels of production, thus further affecting the revenues from the sale of milk.

At least over the short term, water must be entirely purchased because either the livestock have been relocated or because the water supplied to the farm is contaminated. The economic impact of the water quality damage could simply be the purchase of water. Here, it is important to highlight that the recovery of the damage, and thus the perpetuation of the economic impact, may also depend on public action. The longer it takes public authorities to restore the water supply, the longer farmers must buy and supply the water.

Thus, in the case of floods, the impact on animal welfare also depends also on the timely action of public authorities. Therefore, the need for a holistic approach to animal welfare, which involves balancing animal-based direct measures with environmental factors and farm management variables under extreme conditions, seems suitable for the “flooded farm” scenario.

In the case of natural disasters, it is critical to assess the level of animal welfare of a herd [24]. Animal welfare assessments are poorly applied in situations where the environment of a farm is dramatically altered, and farmers must make extraordinary management decisions. Nevertheless, the welfare of dairy cows should be a priority to prevent economic losses in the long term.

This study primarily shows that the literature on flood impacts provide relevant information about variables that should be taken into account to evaluate overall animal welfare in the case of floods. Second, this study proposes a comprehensive analysis of several factors that can occur on farms to evaluate the impact of floods on dairy farms. In situations of flood risk, such analyses can assist farmers in assessing the pre-flood and post-flood conditions of the farm. From this perspective, this study may also provide an approach to develop a simple tool to evaluate damage on dairy herds, emphasizing the importance of data collection for proper technical and economic management of livestock farms. Finally, the results could be relevant to scholars because of the novelty of the approach, and to policy makers who might benefit from the research when designing public interventions for disaster management, especially considering mitigation measures targeting agriculture and, in particular, the livestock farming sector.

## Figures and Tables

**Table 1:** Final sample of papers selected.

Authors	Year	Title	Location	Aim	Method	References
Crist et al.	2020	Flooding on Beef and Swine Farms: A Scoping Review of Effects in the Midwestern United States	USA	“The purpose of this scoping review was to identify the potential impacts of flooding on beef and swine farms in the Midwest US and to identify knowledge gaps related to those impacts”	Review	[29]
Rahman et al.	2020	Seroprevalence and distribution of leptospiral serovars in livestock (cattle, goats and sheep) in flood-prone Kelantan, Malaysia	Asia	“To determine the serological prevalence of leptospiral infection in livestock after a voluminous flood in 10 districts of the Malaysian state of Kelantan”	Case study	[30]
Gaviglio et al.	2019	Evaluating the flood damage on dairy farms: a methodological proposal	Europe	“The purpose of this study is to develop a conceptual model for the assessment of the economic damages of floods on dairy farming systems”	Survey	[9]
Escarcha et al.	2018	Understanding climate change impacts on water buffalo production through farmers’ perceptions	Asia	“To understand how farmers perceive climate change risks and impacts on their water buffalo production systems, and how these risk perceptions inform farmers’ decisions to make changes to their production systems to respond and adapt to climate change”	Survey	[18]
Bissett et al.	2018	Preparation and Response for Flooding Events in Beef Cattle	USA	To synthesize strategies to prepare and respond to the impact of flood events on beef cattle farms	Review	[31]
Waggoner et al.	2018	Feeding and Watering Beef Cattle During Disasters	USA	“The objective of this article is to provide a general overview of feeding, watering, and managing beef cattle following select natural disasters or emergency situations”	Review	[32]
Ijaz et al.	2018	Sero-epidemiology and haemato-biochemical study of bovine leptospirosis in a flood-affected zone of Pakistan	Asia	To investigate the seroprevalence and associated risk factors for bovine leptospirosis in a flood-affected zone of Punjab, Pakistan	Case study	[33]
Maksimovic et al.	2017	Apparent role of climate change in a recent anthrax outbreak in cattle	Europe	“To describe an anthrax outbreak in cattle that coincided with climate and weather changes”	Case study	[34]
Wasiński et al.	2012	Occurrence of leptospirosis in domestic animals reared on exposed or non-exposed to flood areas of eastern Poland	Europe	“To investigate occurrence of *Leptospira* sp. in swine and cattle reared in the territories of two rural communities of the Lubelskie province, eastern Poland. One of these (A) is situated in the western part of the province near the Vistula River. The mentioned area is often exposed to the impacts of raised levels of the river (wet soil and inundation) but in summer 2010, it was affected by two large floods. By contrast, community “B” is situated in the central part of the province, and it does not experience floods.”	Case study	[35]
Huang et al.	2012	The utilization of commercial soil nucleic acid extraction kit and PCR for the detection of *Clostridium tetanus* and *Clostridium chauvoei* on farms after flooding in Taiwan	Asia	“To apply a combination of a commercial nucleic acid extraction kit and PCR to assess the prevalence of *Clostridia* spp. in soil and to compare the positivity rates for farms before and after flood”	Case study	[36]
Posthumus, et al.	2009	Impacts of the summer 2007 floods on agriculture in England	Europe	“As a case study, this paper reports on the findings of a survey to identify and evaluate the nature, magnitude and distribution of economic impacts of the summer 2007 flood events in rural areas on land-based activities”	Survey	[37]

**Table 2:** Direct flood damages to the herd.

	Damages to the Herd Structure			2 Months	1 Year	3 Years	References
	Flooding Damage that Affects Herd Status						
Surviving animals (a)	Health	Diseases	Mastitis	**	*		[9,37]
			Lameness	**	*		[31,37]
			Other diseases	**	*		[29,30,31,33,34,35,36]
		Injuries		**			[9,29,31]
	Malnutrition	Feed	Feed quality	*	*		[32,37]
		Water	Water quality	*			[37]
	Reproductive efficiency		Conception	*	*		[29]
			Abortion	*	*		
	Stress						[9,29]
	Flooding damage that can cause involuntary culling and other flood-related causes of death						
Dead animals (b)	Health	Diseases	Mastitis		*	*	[9]
			Lameness		*	*	[9]
		Injuries			*	*	[9]
		Other diseases			*		[31]
	Drowning			**		*	[9,37]
	Reproductive efficiency		Conception		*		[9]
			Abortion		*		[9]

** refers to the higher predominance of occurrence compared to that over the medium- and long-terms. * refers to the higher predominance of occurrence compared to that over the long-term.

**Table 3:** Dimension of the damage on the herd.

	Damages to the Herd Structure			2 Months	1 Year	References
Surviving animals	Health	Disease	Mastitis (SCC)		1.5 times more with mastitis	[38]
			Lameness		1.3 times more with lameness	[39]
			Bacterial disease		2.7 times more likely to acquire leptospiral infections	[30]
		Injuries				
	Malnutrition	Feed	Feed quality		Up to 100% of feed to be replaced	[32]
		Water	Water quality	Up to 100% of water to be replaced		[32]
	Reproductive efficiency		Conception			
			Abortion			
